# Molecular Docking and Experimental Analysis of Essential Oil-Based Preparations on Biofilm Formation on Orthodontic Archwires

**DOI:** 10.3390/ijms252413378

**Published:** 2024-12-13

**Authors:** Vlad Tiberiu Alexa, Aurora Doris Fratila, Roxana Oancea, Atena Galuscan, Octavia Balean, Vanessa Bolchis, Berivan Laura Rebeca Buzatu, Diana Obistioiu, Mukhtar Adeiza Suleiman, Daniela Jumanca

**Affiliations:** 1 Clinic of Preventive, Community Dentistry and Oral Health, Victor Babes University of Medicine and Pharmacy, Eftimie Murgu Sq. no 2, 300041 Timisoara, Romania; vlad.alexa@umft.ro (V.T.A.); galuscan.atena@umft.ro (A.G.); balean.octavia@umft.ro (O.B.); vanessa.bolchis@umft.ro (V.B.); berivan.haj-abdo@umft.ro (B.L.R.B.); jumanca.daniela@umft.ro (D.J.); 2 Translational and Experimental Clinical Research Center in Oral Health (TEXC-OH), Department of Preventive, Community Dentistry and Oral Health, Victor Babes University of Medicine and Pharmacy 14A Tu-dorVladimirescu Ave., 300173 Timisoara, Romania; 3 Faculty of Dental Medicine, Ludwig-Maximilian-University Munich, Goethestraße 70, 80336 München, Germany; a.fratila@campus.lmu.de; 4 Faculty of Agriculture, University of Life Sciences “King Michael I” from Timisoara, Calea Aradului 119, 300645 Timisoara, Romania; dianaobistioiu@usvt.ro; 5 Faculty of Life Science, Department of Biochemistry, Ahmadu Bello University, Zaria 810107, Kaduna State, Nigeria; masuleiman@abu.edu.ng

**Keywords:** essential oils, molecular docking, dentistry, dental health, oral diseases, orthodontics, antibacterial activity

## Abstract

Good oral hygiene is crucial during treatment with fixed appliances, emphasising the need for additional or alternative oral health methods during orthodontic treatment. This study investigates the effect of essential oil (EO)-based preparations on biofilm adhesion to orthodontic archwires. Five identical-sized orthodontic archwires of different materials were tested using therapeutic and preventive applications of essential oils. This study also used molecular docking to explore how essential oil compounds interact with key proteins of common oral pathogens like *Staphylococcus aureus* and *Streptococcus mutans*. We found that the constituent materials heavily influence the antimicrobial effects of essential oils on different orthodontic archwires. Stainless steel-based orthodontic archwires demonstrated the highest efficacy in antimicrobial protection against *S. mutans* strains (maximum BIP = 28.82% on the epoxy-coated SS). Conversely, inhibition effects in preventive applications against *S. aureus* were observed exclusively with titanium–molybdenum alloy orthodontic archwires across all tested emulsions (maximum BIP = 29.44%). CuNiTi alloys showed ineffectiveness in preventive treatments, as none of the EO mixtures inhibited biofilm development on this material. After biofilm contamination with *S. mutans* and *S. aureuss* strains, the ternary emulsion was most effective for four out of five orthodontic archwires. Computational analysis revealed strong binding interactions between essential oil compounds and key proteins of *S. aureus* and *S. mutans*, highlighting specific amino acid residues that are critical for these interactions. Based on the results, stainless steel with epoxy coating or TMA archwires, combined with BEO/CEO/OEO ternary mixture, are recommended for optimal antibacterial protection against biofilm formation on orthodontic archwires.

## 1. Introduction

To achieve optimal dental alignment and aesthetics, orthodontic treatment is often required. Archwires are an essential part of the devices used in fixed orthodontic therapy for facilitating tooth movement. The choice of archwire material holds significance not only for biomechanical efficiency but also for its influence on oral health [1,2,3]. One key concern is the accumulation of biofilm, a complex microbial community that forms on surfaces within the oral cavity [4]. This biofilm can lead to oral health issues such as caries and periodontitis, potentially complicating fixed orthodontic therapy by increasing the risk of enamel demineralization, periodontal disease, and overall treatment inefficacy [5,6,7,8]. Effective prevention and management strategies are essential to mitigate these risks commonly encountered in these patients and ensure optimal oral health outcomes during orthodontic treatment [9,10]. The quantity and type of biofilm buildup can be influenced by the material of the archwire, which has an effect on patient oral hygiene, treatment results, and overall oral health [11].

Therefore, research into the relationship between biofilm formation and materials used during orthodontic treatment is essential for enhancing treatment strategies and patient well-being. This article explores the crucial relationship between archwire materials and biofilm development, illuminating the impact of archwire material choice on oral health during orthodontic treatment. Essential oils (EOs) play a significant role in microbiology, providing an alternative to synthetic antimicrobials and contributing to the development of new therapeutic and preventive strategies [12,13].

In this study, we focused on orthodontic archwires due to their direct and significant role in biofilm formation during orthodontic treatment. Archwires are constantly exposed to oral fluids, which makes them highly susceptible to microbial colonisation. As archwires exert continuous force on the teeth, they are prone to microbial adhesion, particularly in areas that are harder to clean, such as near brackets or beneath the archwire itself. Their surface properties significantly influence microbial attachment, making them an ideal focus for studies on biofilm formation and antimicrobial interventions [14]. Additionally, archwires are often exposed to various treatments or coatings (such as epoxy coatings), which can alter their surface characteristics and biofilm resistance properties. While brackets and other orthodontic materials also influence microbial adhesion, archwires are often more prone to biofilm formation due to their continuous presence and surface characteristics, such as roughness and the presence of coatings [15]. Thus, studying the antimicrobial effects of natural essential oils on different types of archwires, which are among the most commonly used during fixed orthodontic therapy, is necessary for improving orthodontic outcomes and minimising the risk of biofilm-related complications.

Advancements in developing more qualitative orthodontic archwire materials for better treatment outcomes have significantly increased recently, with doctors now having a wide range of materials to choose from during orthodontic treatment [16]. These material innovations have resulted in enhanced properties such as increased super elasticity, thermal shape memory, improved corrosion resistance, and better biocompatibility [17,18].

A study by Tawfik et al. (2020) investigated biofilm adhesion on four types of orthodontic archwires [19]. Results showed that copper–nickel–titanium (Cu-NiTi) alloys demonstrate antimicrobial properties, likely due to copper’s antibacterial effects, suggesting that Cu-NiTi alloys may have reduced microbial adherence compared to other types of orthodontic archwires. [20]. Developed by Charles Burstone in the 1980s, the Beta Titanium Alloy (TMA) is a stabilised beta-phase titanium alloy composed of titanium (79%), molybdenum (11%), zirconium (6%), and tin (4%). TMA wires have shown increased biofilm adhesion due to their higher surface roughness than other alloys. This characteristic can enhance bacterial retention and biofilm formation, particularly in long-term use. On the other hand, Nickel–Titanium Alloy (NiTi) wires typically exhibit lower biofilm adhesion than TMA due to their smoother surfaces and enhanced corrosion resistance, making them effective at minimising bacterial attachment during orthodontic treatment [19]. Regarding biofilm adhesion to stainless steel (SS) archwires, Eliades et al. (1995) found that stainless steel exhibits the highest critical surface tension and energy, which contributes to a greater plaque retention capacity, and it also induces changes in the oral environment, such as decreased pH and increased plaque accumulation [21].

The desire for more aesthetically pleasing orthodontic equipment has increased due to the rise in adult orthodontic patients. Aesthetic polymer-based wires and metallic archwires covered with polymer materials both emerged in response to the desire for more aesthetically pleasing materials [22]. Studies show mixed results regarding their effect on biofilm adhesion. Some studies indicate that the coating may not significantly reduce biofilm formation and can occasionally lead to increased bacterial attachment due to surface roughness variations introduced by the coating. Taha et al. (2016) explored different coated wires, noting variability in bacterial adhesion based on coating types and roughness [23]. On the other hand, coatings on orthodontic metal wires may affect mechanical features, such as bending behaviour, as well as surface attributes, roughness, and hardness [11,24]. Orthodontic archwires serve as a substrate for dental plaque accumulation, thereby increasing the microbial load within the oral cavity [25,26,27]. It has been discovered that a number of orthodontic archwire surface properties, including surface roughness, surface free energy, corrosion susceptibility, etc., have an impact on the adherence of biofilm and microbial colonisation [11]. Few studies have been conducted to assess and compare the biofilm adherence of various archwire materials despite the vast research on the mechanical and physical characteristics of orthodontic archwires.

This study aims to research the effect of natural preparations based on essential oils (EOs) against biofilm adhesion using five different orthodontic archiwires materials with the same dimensional characteristics, respectively:

1. Aesthetic tooth-colored epoxy-coated stainless steel;
2. Copper–nickel alloy (Cu-NiTi);
3. Titanium–molybdenum alloy (TMA);
4. Stainless steel (SS);
5. Nickel–titanium alloy (NiTi).

The experiment was carried out with two distinct approaches regarding the use of essential oils:

1. For a preventive effect, orthodontic archwires were first immersed in natural preparations obtained according to the methodology described in our previous research [28] and then introduced into culture medium with *Staphylococcus aureus* and *Streptococcus mutans* strains [29].
2. For a therapeutic effect, orthodontic archwires with bacterial load were treated with emulsions based on essential oils, and the effect was measured at different time intervals.

The second goal of this study was to assess how the compounds of essential oils interact with key proteins of pathogenic bacteria such as *S. aureus* and *S. mutans* bacteria, which commonly develop in the oral cavity [30,31,32]. Molecular docking is one of the rational and computer-aided tools used to validate classical laboratory experiments and reduce the time required to understand the possible interactions between identified compounds of oils and proteins of bacteria, particularly, key proteins involved in the pathogenesis of the aforementioned microorganisms [33,34,35].

## 2. Results

### 2.1. Results Regarding the Preventive Antimicrobial Effect of EOs on Orthodontic Archwires

**Figure 1:**
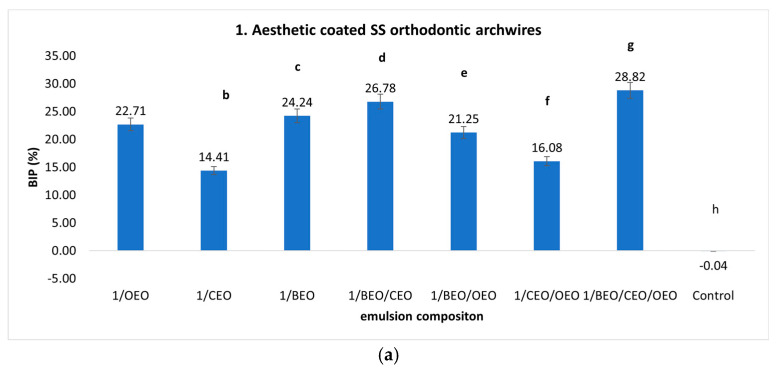

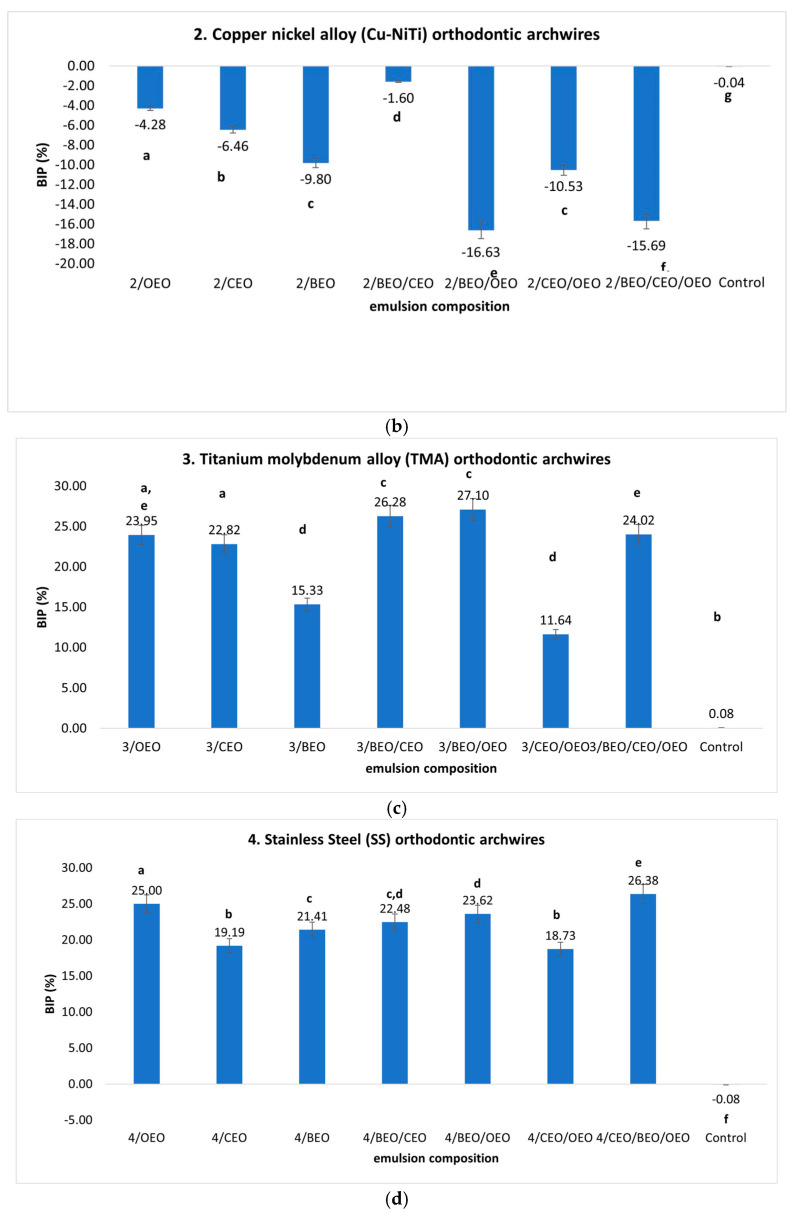

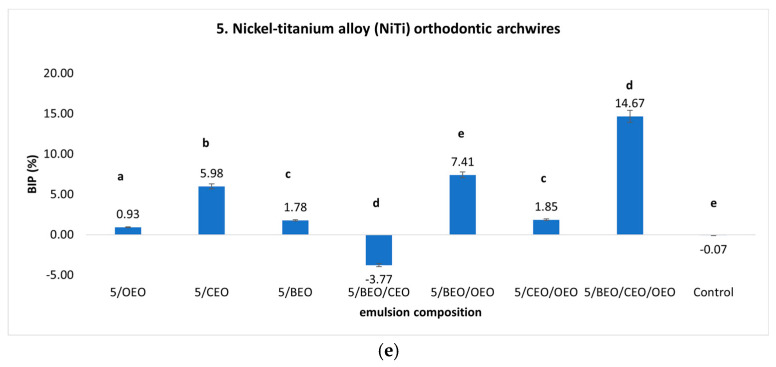
(**a**–**e**) BIP (%) values for preventive treatment against *S. mutans* strains on orthodontic archwires using natural preparation based on Eos. (Different letters in columns indicate significant differences between values according to *t*-test; *p* < 0.05. Superscript letters assigned to columns represent these differences).

Figure 1 illustrates the BIP against *S. mutans* strains observed on five orthodontic archwires pre-treated with an emulsion based on essential oils (EOs). The results demonstrate positive BIP values for four out of the five orthodontic archwires tested. This finding underscores the protective and inhibitory effects on microbial growth achieved through the preventive administration of treatments based on natural preparations containing essential oils. A negative inhibition rate (−1.60 to −16.63%) was obtained for all emulsions tested using the copper–nickel–titanium (Cu-NiTi) alloy, and negative and minimal positive inhibition rates were obtained for the NiTi alloy.

Positive values for the *S. mutans* inhibition rate, indicative of antimicrobial activity, were recorded using monocomponent, binary, and ternary emulsions for the preventive treatment of orthodontic arches composed of stainless steel and the titanium–molybdenum alloy. Thus, for aesthetic tooth-coloured epoxy-coated stainless steel orthodontic archwires (Figure 1a), the inhibition rate of *S. mutans* development ranged from 14.41%, which was the minimum value recorded for the CEO emulsion, to 28.82%, which was the maximum value observed with the ternary mixture of BEO/CEO/OEO.

Regarding the monocomponent emulsions, it was observed that the maximum inhibition rate was obtained for the natural preparation based on bergamot, followed by oranges and cloves. The binary mixture of BEO/CEO resulted in synergistic effects, with a BIP of 26.78%, which is higher than the values recorded for the monocomponent emulsions (24.24% for BEO and 14.41% for CEO). On the contrary, the BEO/OEO binary emulsion exerts antagonistic effects, reducing the antimicrobial capacity against *S. mutans* strains (BIP = 21.25%) compared to the monocomponent emulsions (BIP OEO = 22.71%, BIP BEO = 24.24%). The CEO/OEO association in the binary emulsion (BIP = 16.08%) inhibited the antimicrobial effect recorded by OEO in the monocomponent emulsion (BIP = 22.71%). However, it favoured the activity exerted by CEO (BIP = 14.41%), registering intermediate values regarding the inhibition rate of *S. mutans* strains compared to the values obtained in the case of monocomponent emulsions. The maximum antimicrobial effect against *S. mutans* was recorded using the ternary mixture based on BEO/CEO/OEO (BIP = 28.82%). Statistically significant differences were recorded for all samples that were analysed.

For orthodontic archwires made of Cu-NiTi (Figure 1b), the essential oil-based emulsions analysed did not exhibit preventive effects in reducing the biofilm generated by *S. mutans*. The inhibition rates were negative for all the samples analysed, with BIP values ranging from −1.6% to −16.63%. The highest inhibition rate was observed with the BEO/OEO binary mixture.

Titanium–molybdenum alloy (TMA) orthodontic archwires ensured an inhibition rate against *S. mutans* strains between 11.64 and 27.10%, with maximum values in the case of the BEO/OEO binary mixture (Figure 1c). The ternary mixture also provides an excellent preventive effect (BIP = 24.02%).

Stainless steel (SS) orthodontic archwires exerted a positive inhibition effect similar to that observed with aesthetic-coated SS orthodontic archwires. Notably, SS archwires were more responsive to monocomponent emulsions based on OEO and CEO, exhibiting a superior inhibition rate. In contrast, the aesthetic coated SS archwires demonstrated a better response to BEO as a monocomponent antimicrobial agent (Figure 1d). When using stainless steel (SS) orthodontic archwires, there were insignificant differences in the antimicrobial effects against *S. mutans* when different monocomponent essential oils (EOs) or their mixtures were used. The lowest inhibition value (BIP = 19.19%) was also obtained in this case for the CEO, followed by BEO (21.41%) and OEO (25.00%). For the BEO/CEO mixture, there was a synergistic effect of a slight increase in the inhibition rate (BIP = 22.48%) compared to the monocomponent emulsions. For the BEO/OEO mixture, the inhibition effect was between that obtained for the monocomponent emulsions (BIP = 23.62%). Meanwhile, for the CEO/OEO mixture, the effect was antagonistic, reducing inhibition (BIP = 18.73%). Instead, combining all three EOs analysed in the ternary emulsion led to the maximum inhibition effect on *S. mutans* (BIP = 26.38%).

For the alloys based on NiTi, the recorded BIP values were positive (except for the use of the binary emulsion based on BEO/CEO—BIP = −3.77%); the inhibition rate values obtained were much lower than those recorded in the case of steel-based orthodontic archwires (Figure 1e). In this case, we observe that OEO and BEO inhibited the development of *S. mutans* to a lesser extent than CEO, in opposition to the effect obtained in the case of steel base orthodontic archwires. However, synergistic effects of increasing the inhibition rates were observed with the BEO/OEO combination (BIP = 7.41%) but antagonistic with the BEO/CEO combination (BIP = −3.77%). This time, the maximum inhibition effect was also obtained in the case of the ternary emulsion (BIP = 14.67%).

**Figure 2:**
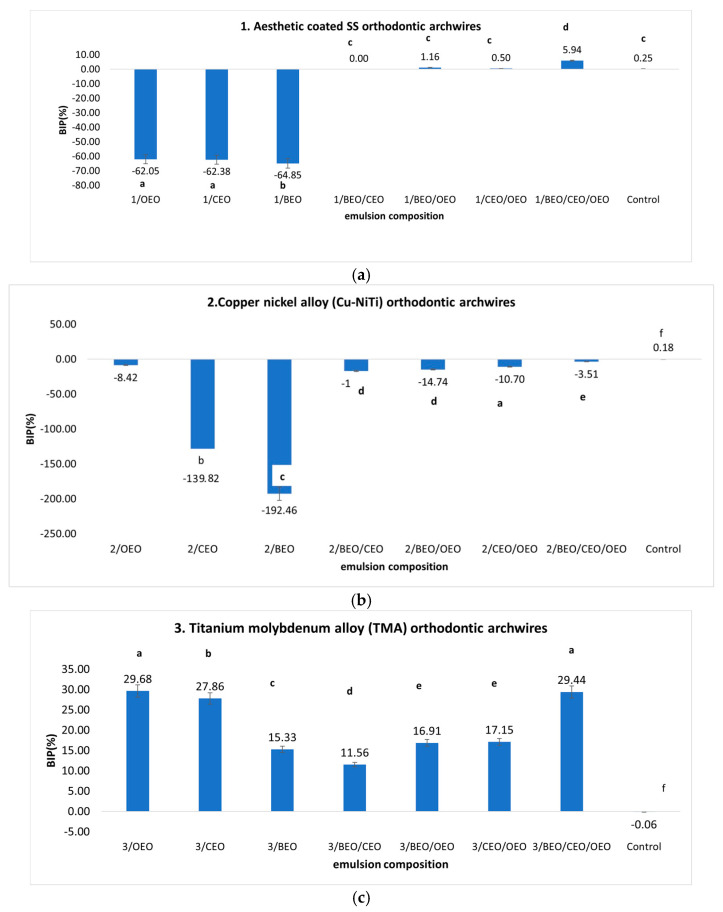

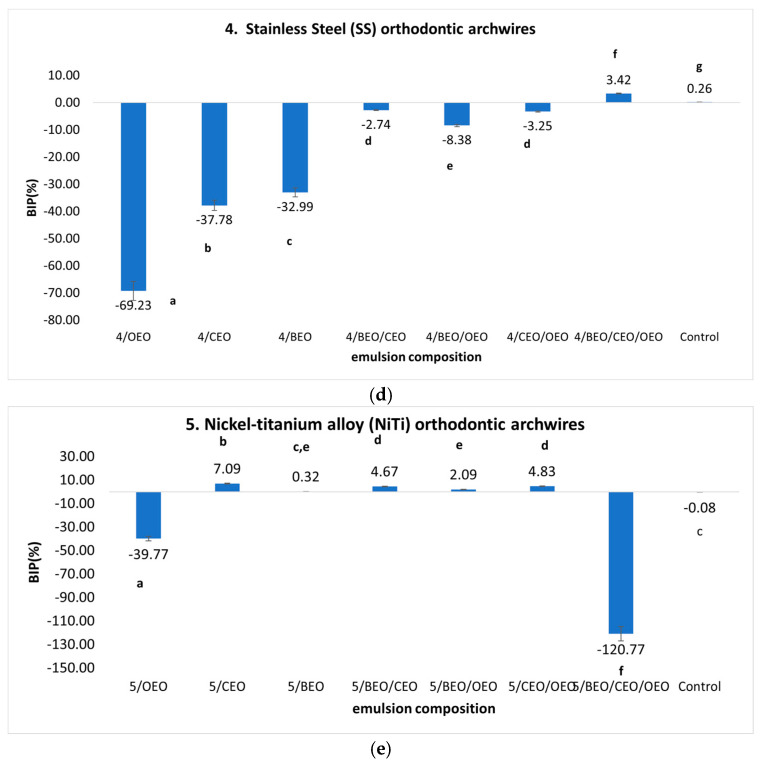
(**a**–**e**) BIP (%) values for preventive treatment against *S. aureus* strains on orthodontic archwires using natural preparation based on Eos. (Different letters in columns indicate significant differences between values according to *t*-test, *p* < 0.05. Superscript letters assigned to columns represent these differences).

Figure 2 illustrates the preventive antimicrobial protection provided by natural preparations based on essential oils (EOs), focusing on their effects on *S. aureus* strains deposited on orthodontic archwires of different materials.

Only the orthodontic archwires based on titanium–molybdenum alloy (TMA) showed antimicrobial protection when they were pre-treated with EO-based emulsions, with inhibition rates between 11.56% and 29.68%.

The aesthetic coated SS orthodontic archwires (Figure 2a), when pre-treated with monocomponent emulsions (OEO, BEO, CEO), promoted the development of the microfilm generated by *S. aureus* strains, with a positivity rate ranging between 37.97% and 35.15%. Using binary mixtures exerted synergistic effects due to the complementary chemical composition of EOs, which counteracts the potentiation effect. However, the beneficial effects of bacterial inhibition were poorly observed (BIP between 0 and 5.94%), and the maximum value was obtained when using the ternary mixture (BEO/CEO/OEO).

In the context of the preventive application of emulsions based on essential oils (EOs) on orthodontic archwires fabricated with Cu-NiTi alloy (Figure 2b), the phenomenon of potentiation in the development of the bacterial biofilm of *S. aureus* was notably accentuated. Negative BIP values were recorded for all applied emulsions, with maximum values in the case of using the CEO monocomponent emulsion (BIP = −192.46%). Lower potentiation effects were recorded when using binary mixtures (BIP between −10.7 and 16.84%), in addition to ternary mixtures (BIP = 3.51%).

Alloys based on TMA (Figure 2c) exhibited optimal efficacy in inhibiting the development of *S. aureus* when subjected to preventive treatment with natural preparations based on essential oils (EOs). The inhibition rate varied between 11.56 and 29.68%. In this case, OEO was the most efficient EO, offering maximum protection, but the ternary mixture also registered a good inhibition rate of 29.44%.

In the case of steel-based (SS) archwires (Figure 2d), there was an observed potentiation of bacterial growth caused by *S. aureus* strains, particularly notable with single-component emulsions. The potentiation rate increased in the following order: BEO < CEO < OEO. A slight protective effect was recorded in the case of the ternary mixture with a minimum inhibition rate of 3.42%.

The alloy based on NiTi (Figure 2e) showed low positive inhibition rates in binary monocomponent mixtures based on CEO and BEO (BIP = 0.32–7.09%). In contrast, the emulsion based on OEO and the ternary mixture BEO/CEO/OEO showed negative inhibition rates for *S. aureus* between −39.77% and 120.77%.

**Figure 3:**
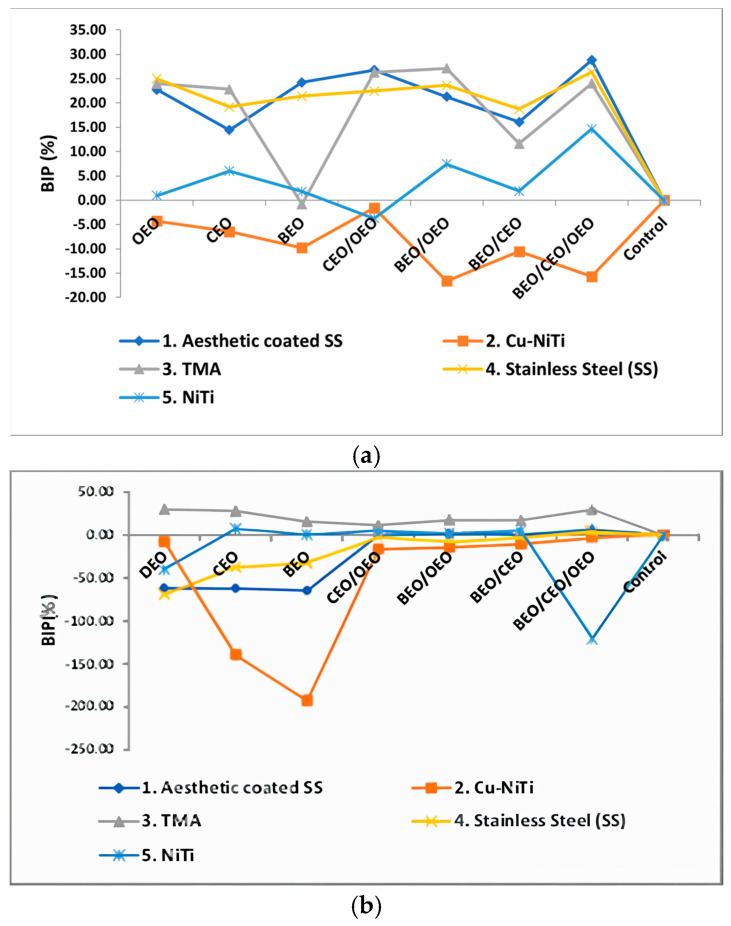
BIP (%) values for preventive treatment against *S. mutans* (**a**) and *S. aureus* (**b**) and strains on different orthodontic archwires using natural preparation based on EOs.

A comprehensive assessment of the bacterial inhibition rate against *S. mutans* (a) and *S. aureus* (b) strains revealed higher positive values against *S. mutans* and lower values against *S. aureus* strains following preventive treatment with essential oil (EO) emulsions (Figure 3). Regarding the orthodontic materials utilised, a distinction was observed in inhibiting microbial biofilm development between steel-based orthodontic archwires (SS and aesthetically coated SS) and those based on NiTi for both bacterial strains analysed. Notably, the TMA archwires demonstrated enhanced inhibition of *S. aureus* strain development compared to *S. mutans*. Regarding the type of emulsion utilised for prevention, the most favourable outcomes for both investigated bacterial strains were achieved with the ternary mixture.

### 2.2. Results of Therapeutic Antimicrobial Assay on Orthodontic Archwires Using Essential Oils (EOs)

**Figure 4:**
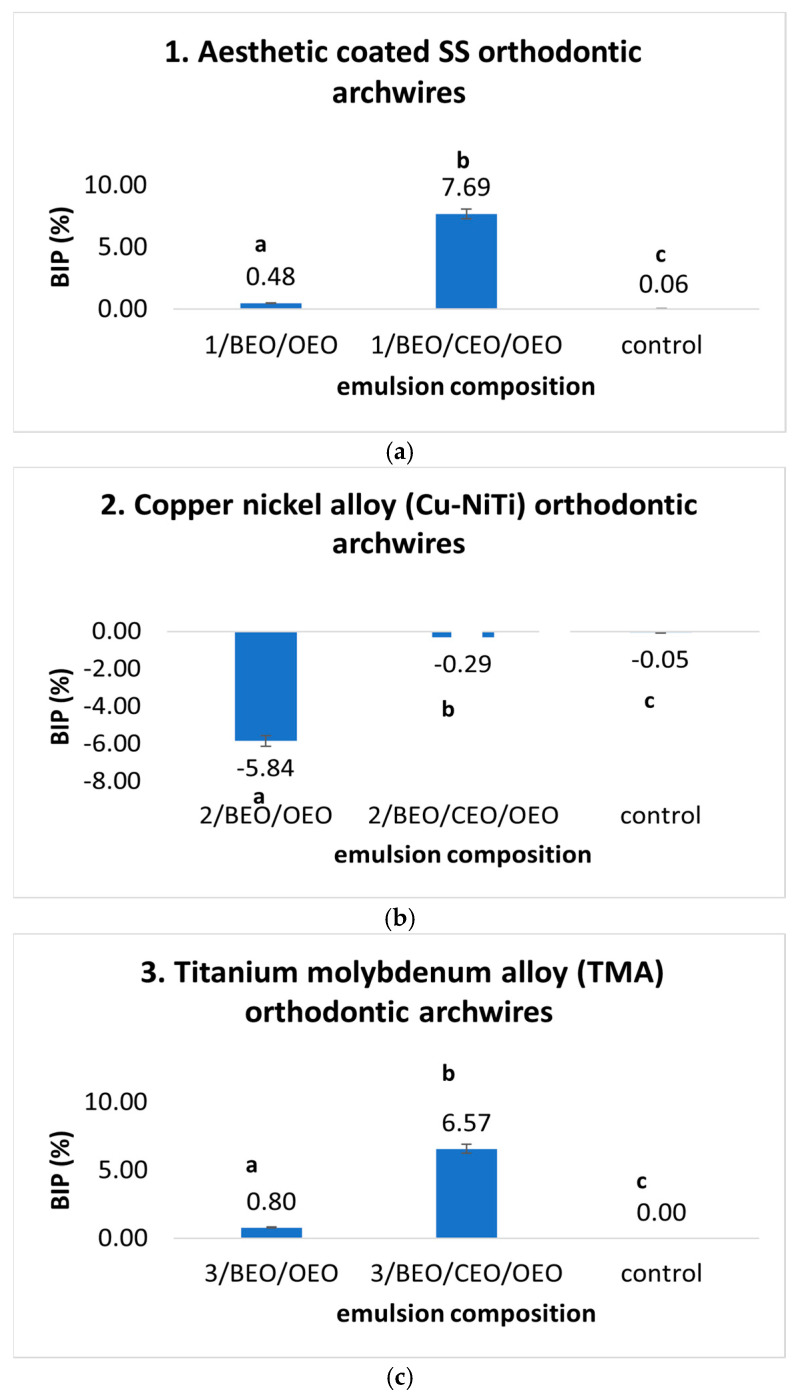

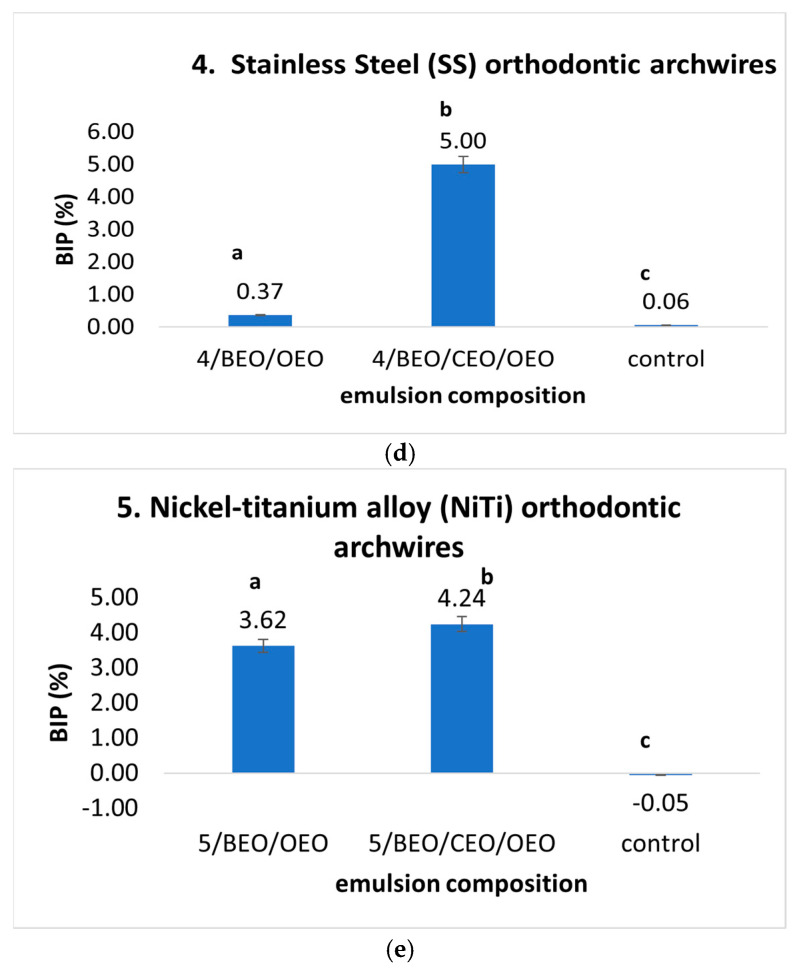
(**a**–**e**) BIP (%) values for curative treatment against *S. mutans* strains on orthodontic archwires using natural preparation based on Eos. (Different letters in columns indicate significant differences between values according to *t*-test, *p* < 0.05. Superscript letters assigned to columns represent these differences).

**Figure 5:**
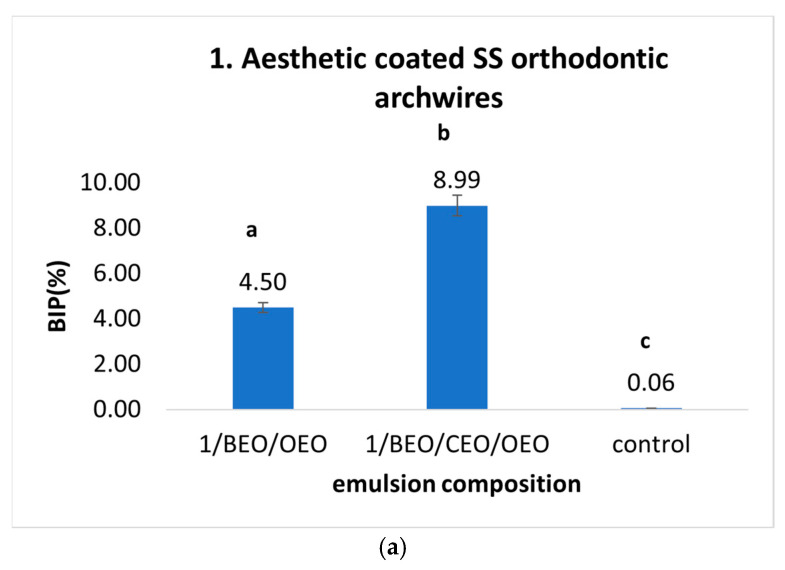

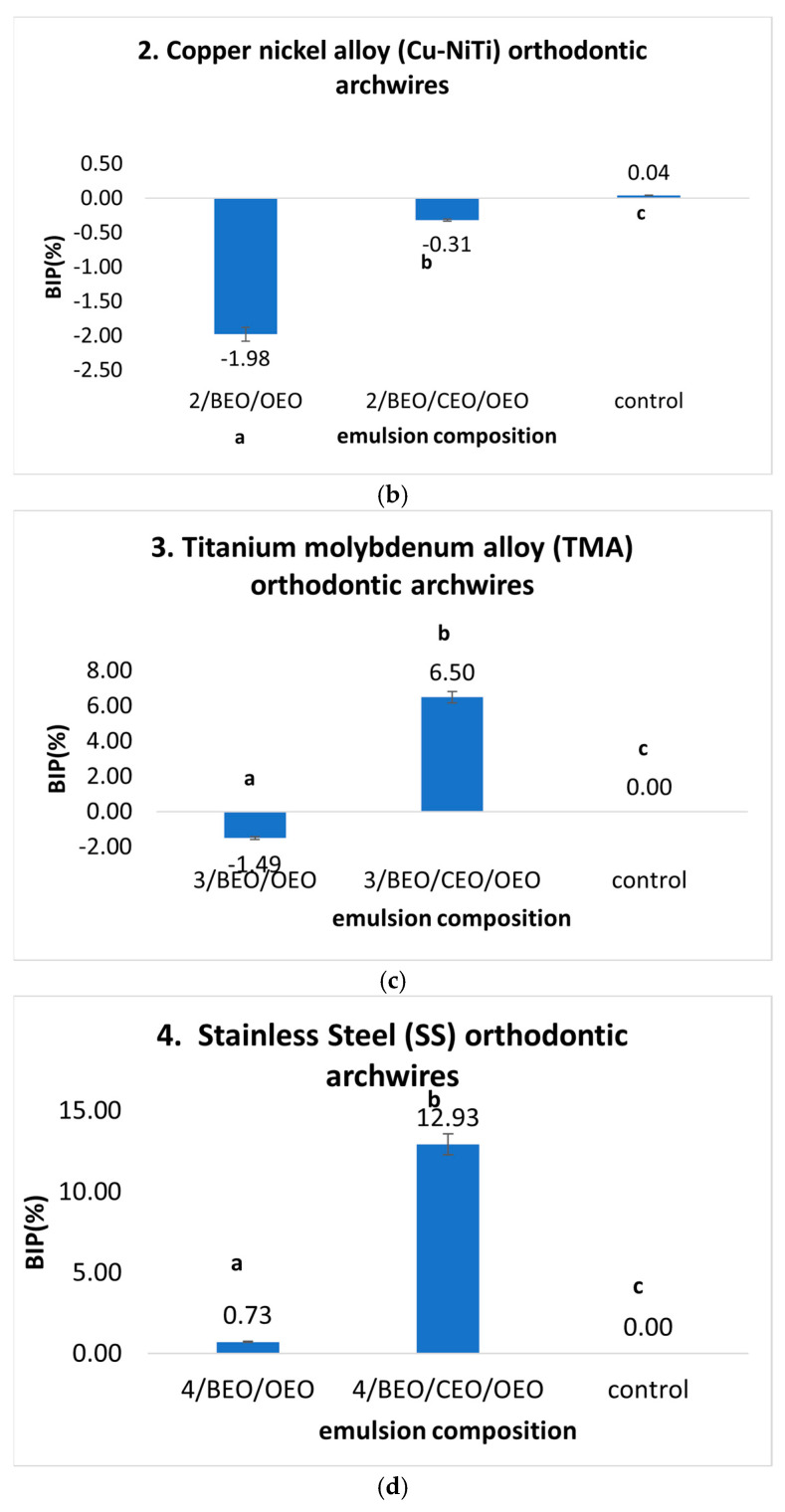

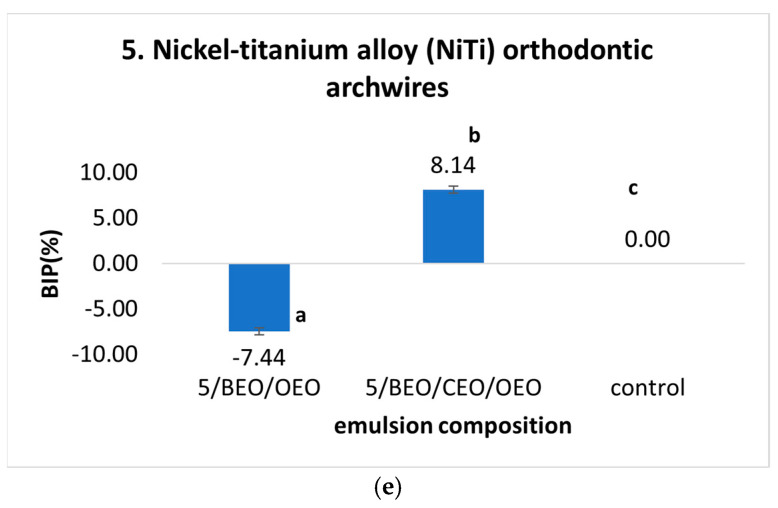
(**a**–**e**) BIP (%) values for curative treatment against *S. aureus* strains on orthodontic archwires using natural preparation based on EOs. (Different letters in columns indicate significant differences between values according to *t*-test, *p* < 0.05. Superscript letters assigned to columns represent these differences).

Using natural compounds as a treatment modality demonstrated significant antimicrobial activity against bacterial strains, specifically *S. mutans* (Figure 4) and *S. aureus* (Figure 5). This confirms their antimicrobial effects in binary and ternary formulations, further supporting their preventive capabilities. Thus, statistically significant differences were observed in the case of using ternary mixtures compared to binary ones based on BEO/OEO and compared to the control. A positive inhibition rate was recorded in the case of both mixtures tested against *S. mutans* on four of the five tested orthodontic archwires with an inhibition rate between 4.24 and 7.69% for the BEO/CEO/OEO ternary mixture and between 0.37 and 3.62% for the BEO/OEO mixture. On the other hand, orthodontic archwires based on CuNiTi potentiated the development of *S. aureus* in the case of treatment with BEO/OEO solution and BEO/CEO/OEO ternary solution. The results align with those obtained from the preventive experiment, with the inhibition rate of *S. mutans* on various orthodontic archwires treated with ternary emulsions. The inhibition rate on the tested archwires varied in the following order: aesthetic-coated SS > TMA > SS > NiTi > CuNiTi in the therapeutic experiment and aesthetic SS > SS > TMA > NiTi > CuNiTi in the preventive administration.

**Figure 6:**
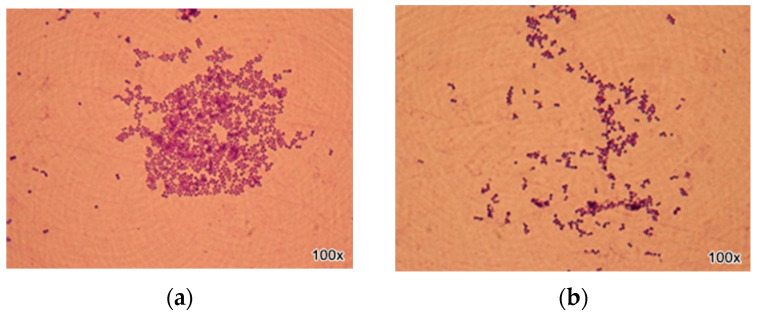
Optical microscopy image of *S. aureus* strains on orthodontic archwires (aesthetic epoxy coated stainless steel archwire) using natural preparation based on EOs (**a**) (without treatment), (**b**) (with BEO/CEO/OEO ternary mixture).

Regarding the inhibition of *S. aureus* by treatment with the OEO/BEO/CEO ternary emulsion and the binary OEO/BEO emulsion (Figure 6), a similar inhibition trend was observed for four types of tested wires, except the CuNiTi-based wire. For the CuNiTi wire, the inhibition rate ranged from 6.50% to 12.93%. On the other hand, the BEO/OEO binary emulsion potentiated the activity of *S. aureus*, except for steel-based orthodontic archwires, where the inhibition rate was 0.73% for the SS archwires and 4.5% for the aesthetic-coated SS archwires. In the case of *S. aureus*, the maximum inhibition rate by treatment was obtained for steel-based orthodontic arches.

**Figure 7:**
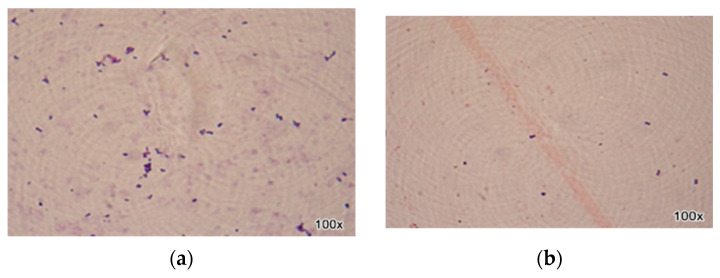
Optical microscopy image of *S. mutans* strains on orthodontic archwires (aesthetic epoxy coated stainless steel archwire) treated with essential oil-based natural preparations (**a**) (without treatment), (**b**) (with BEO/CEO/OEO ternary mixture).

Figure 6 and Figure 7 illustrate the contrast between the analysed species: *Staphylococcus* spp. and *Streptococcus* spp. The images present the effect of the most efficient natural preparation (the ternary mixture) on the most recommended orthodontic archwire (aesthetic epoxy-coated stainless steel archwire). A reduction in the structures of the analysed bacterial strains can be observed, evident in the decrease in Gram-positive cocci arranged in clusters (*Staphylococcus* spp.) and Gram-positive cocci arranged in chains (*Streptococcus* spp.).

**Figure 8:**
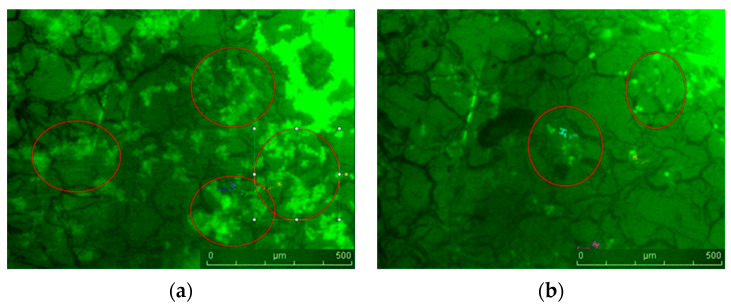
Confocal images before (**a**) and after (**b**) treatment with natural preparation with BEO/CEO/OEO ternary mixture on aesthetic epoxy-coated stainless steel archwire.

**Figure 9:**
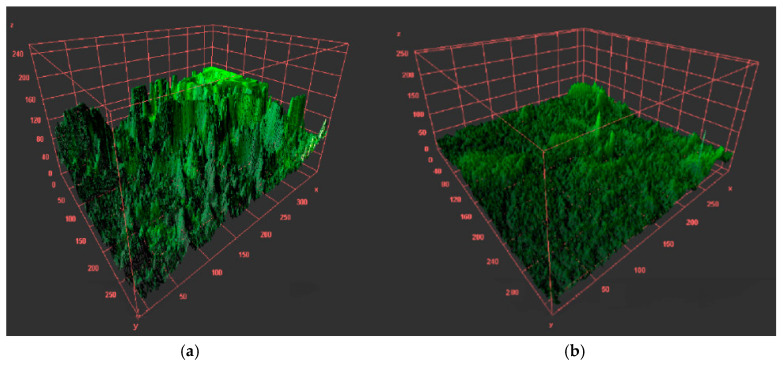
Three-dimensional surface plot of biofilm formation obtained with confocal microscope before (**a**) and after treatment (**b**) with natural preparation with BEO/CEO/OEO ternary mixture on the aesthetic epoxy-coated stainless steel archwire. *X*-axis represents horizontal spatial dimension of biofilm sample, in micrometres. *Y*-axis represents vertical spatial dimension perpendicular to t*X*-axis, in micrometres. *Z*-axis indicates height of microbial biofilm structure, corresponding to density and fluorescence signal intensity of biofilm. Green colouring highlights biofilm regions, and uneven surface suggests spatial variability in biofilm density.

Contaminated and subsequently treated with the most efficacious emulsion, the orthodontic archwires were quantified using confocal microscopy (Figure 8 and Figure 9). It is evident that post-treatment, there was a reduction in microbial load, a phenomenon substantiated by the quantitative findings.

### 2.3. Molecular Docking Assay

Molecular docking analysis was conducted utilising all chemical compounds identified in the three analysed essential oil samples (OEO, CEO, BEO), as documented in Table 1 in our previous paper (Alexa et al., 2019) [28].

**Figure 10:**
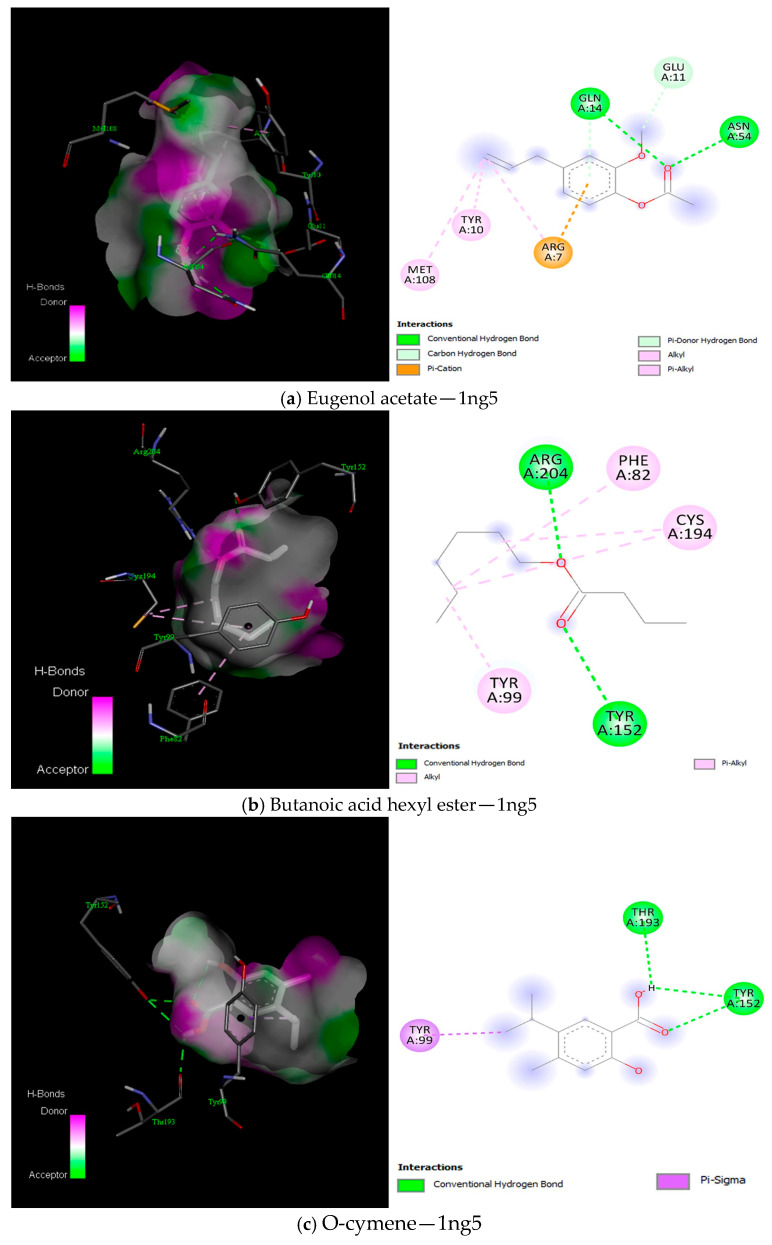
Best binding interaction between compounds (ligands) and 1ng5 protein (3D and 2D) as visualised in Discovery Studio.

**Figure 11:**
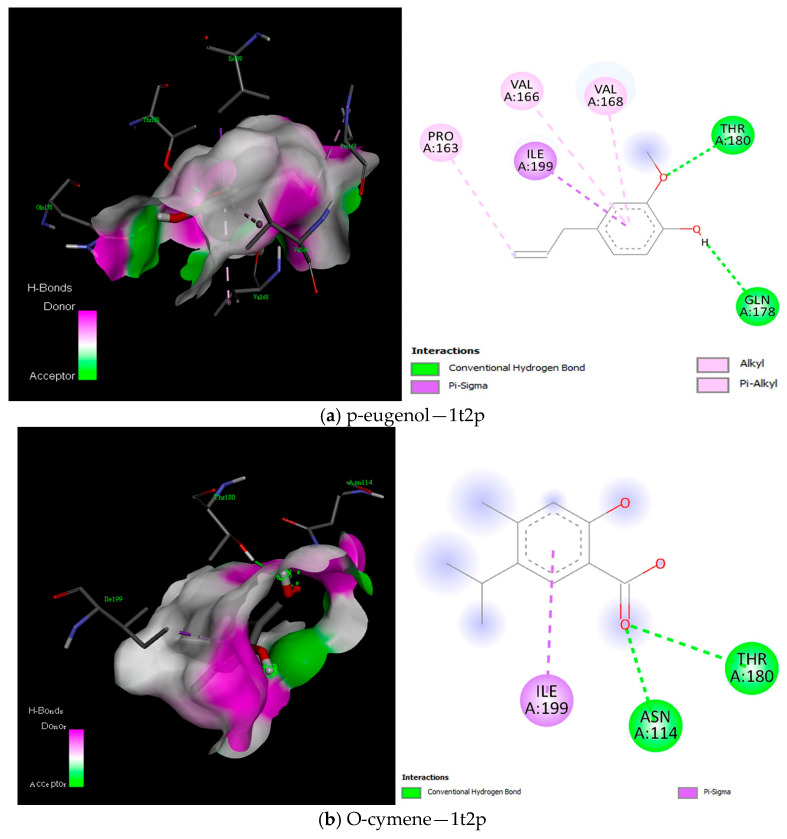
Best binding interaction between compound and 1T2P protein (3D and 2D) as visualised in Discovery Studio.

The docking of ligands (compounds) against Sortase B (1NG5) protein unveiled that Eugenol acetate, Butanoic acid hexyl ester, and O-cymene exhibited the most favourable binding interactions (Figure 10), with Eugenol acetate demonstrating a noteworthy free binding energy of −5.7 kcal/mol (Appendix A). Similarly, the docking of the ligands and Sortase A (1T2P) protein showed that p-eugenol had the best binding interaction with the protein and free binding energy of ≥−5.2 kcal/mol (Figure 11 and Appendix B).

**Figure 12:**
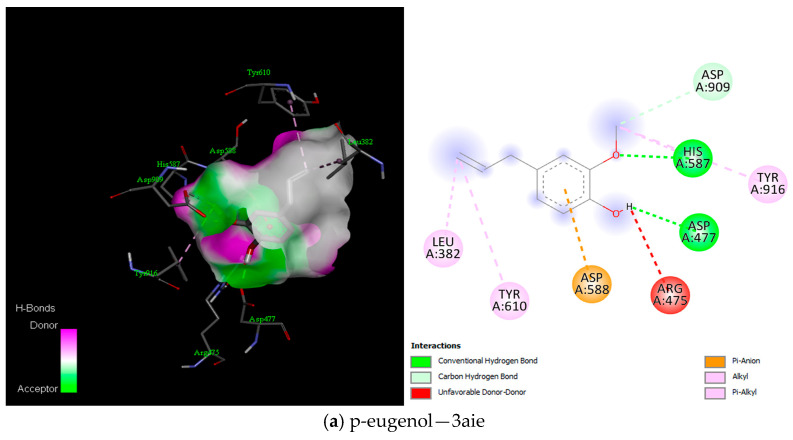

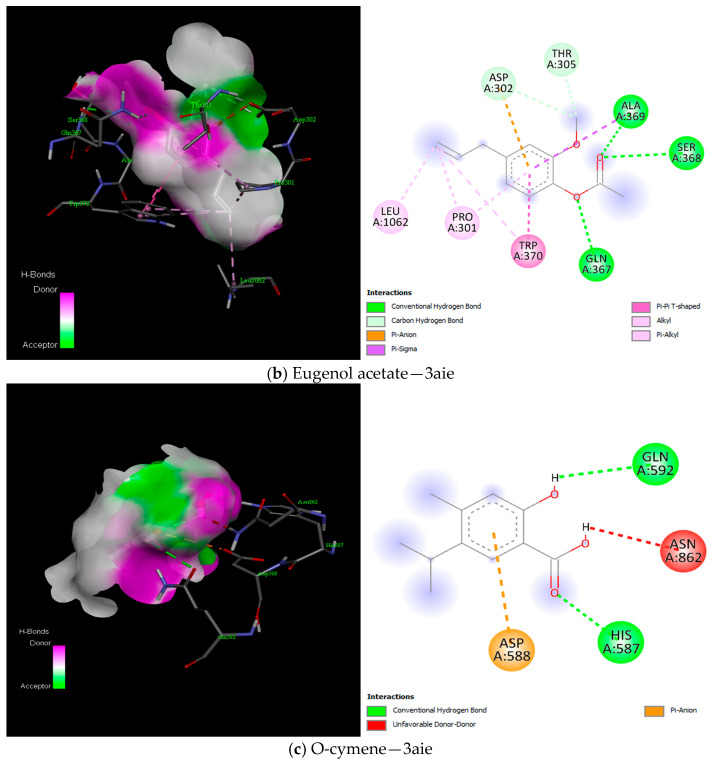
Best binding interaction between compound and 3aie protein (3D and 2D) as visualised in Discovery Studio.

On the other hand, the docking result between the ligands and glucan sucrase (3AIE) also showed that p-eugenol, Eugenol acetate, and O-cymene had a favourable binding interaction with the protein. Eugenol acetate presented the best binding interaction with −6.1 kcal/mol binding energy (Figure 12 and Appendix C).

## 3. Discussion

The craniofacial complex is an extremely elaborate structure influenced by the forces applied during orthodontic treatment. The correct application of orthodontic treatment can affect more than just the craniomandibular system; recent studies prove that changes at the mandibular level can cause anatomical variations at the craniocervical level as well [36]. Thus, the correct use and choice of an optimal orthodontic system from a constructive point of view and of the component materials represent essential elements in orthodontic treatment.

Fixed orthodontic appliances introduce additional surfaces and niches for microbial colonisation, leading to alterations in the oral microbiota [37,38,39]. Studies have demonstrated increased bacterial load, changes in microbial diversity, and shifts in the relative abundance of specific bacterial species during orthodontic treatment. These changes can result in an increased risk of oral diseases, including dental caries and periodontal disease [40,41].

Published research has demonstrated that circumstances that encourage the colonisation and development of *Streptococcus* spp., *Lactobacillus* spp., fungi, and periodontal pathogens increase the accumulation of plaque and stimulate microbial proliferation [42,43,44].

Orthodontic archwires provide an excellent habitat for developing oral microorganisms, especially for strains of *Staphylococcus* and *Streptococcus*, causing periodontal diseases and tooth decay. In the oral cavity, *S. aureus* inhabits the surface of the oral mucosa, saliva, tongue, and periodontal pockets, leading to dentoalveolar infections and oral mucosal lesions [45].

Bergamot, orange, and clove essential oils have demonstrated significant antimicrobial properties, which could play a role in preventing bacterial biofilm formation on orthodontic archwires. Bergamot essential oil, rich in compounds like limonene and linalool, is known to disrupt bacterial membranes by inserting into the lipid bilayer, leading to membrane destabilisation and bacterial leakage, thereby inhibiting the growth of *S. mutans*, a key contributor to oral biofilms on orthodontic appliances [46,47]. Similarly, orange essential oil, containing limonene, exhibits antimicrobial effects by interfering with bacterial membrane integrity, which reduces the adhesion of *Streptococcus* species and other oral pathogens [48]. Clove essential oil, with eugenol as its primary active compound, has potent antibacterial properties through its ability to increase membrane permeability and cause cell death [49,50]. These essential oils, with their hydrophobic components, are particularly effective at targeting bacterial membranes. They could reduce biofilm buildup on orthodontic archwires and improve oral hygiene during orthodontic treatment.

In an attempt to find the optimal solutions for prevention and antimicrobial treatment in the case of patients with fixed orthodontic appliances, the influence of the composite alloys on the biofilm generated by *S. aureus* and *S. mutans* in the oral cavity was followed in two distinct situations: (i) preventive, by applying natural preparations based on EOs, and (ii) application treatment of EOs after microbial contamination.

The evaluation of the preventive effects generated by the use of natural preparations based on EOs as antimicrobial agents against *S. mutans* developed as a biofilm on orthodontic archwires highlights the fact that steel-based orthodontic archwires are less susceptible to contamination if a preliminary application with EOs is carried out compared to archwires based on CuNiTi or NiTi. The inhibition profile was similar for both types of steel-based archwires used. On the contrary, potentiation effects were recorded in the CuNiTi alloy. At the same time, molybdenum-based archwires (TMA) exert inhibition effects comparable to those of steel-based alloys, especially when using ternary mixtures (BEO/CEO/OEO).

The specialised literature presents studies regarding the different microbial loads on orthodontic archwires depending on the nature of the constituent alloy [3,51,52]. This confirms our results regarding the higher susceptibility to microbial loading in alloys based on CuNiTi and NiTi compared to those based on steel, but research on the antimicrobial capacity of EOs in correlation with the type of orthodontic archwires has not been developed so far.

Other authors highlighted that the affinity for biofilm absorbance and concentration depending on the type of archwires varies in order: TMA > Cu-NiTi > aesthetic coated SS [11]. Also, it was observed that among the two analysed strains (*S. mutans* and *S. aureus*), the most abundant strain in the biofilm developed on TMA and SS archwires was *S. mutans*. At the same time, it was observed that CuNiTi orthodontic archwires are more susceptible at contamination with *S. aureus* [51].

These findings are drawn from the results that we obtained, which highlight a lower microbial load with *S. mutans* in the control of steel-based archwires compared to other orthodontic archwires; CuNiTi presented the highest load with *S. aureus* strains. In contrast, in a study conducted by Hepyukselen (2019), CuNiTi archwires demonstrated lower microbial involvement, likely due to the antimicrobial properties of copper [20]. The explanation for this fact could be given by the smoothness of the surface of the steel-based archwire, which does not allow the biofilm to adhere easily [11,53,54].

Also, other studies highlighted that epoxy-coated orthodontic materials are more likely to avoid microbial loading than uncoated ones. This behaviour was attributed to the difference between their surface characteristics, such as surface energy and roughness [55]. Furthermore, the aesthetic coating of wires may provide an added advantage for patients with nickel allergies [54,56]. However, this finding is inconsistent with the results reported by Slonik et al. (2022), who concluded that the available data are insufficient to ascertain whether aesthetic coatings reduce bacterial adhesion [2]. On the other hand, in contrast to our results, Costa Lima et al. (2019) showed that aesthetically coated archwires exhibited increased microorganism adhesion [57]. However, they used coated nickel–titanium archwires compared to our study, in which coated stainless steel was used instead. The extent of bacterial adhesion is significantly influenced by the type of coating material and its surface properties, especially surface roughness and apparent surface energy [58]. Coating treatments should aim to reduce the surface roughness of materials to enhance wire sliding [59]. It is essential to thoroughly assess the benefits and disadvantages of these aesthetic coatings, including their impact on biofilm adhesion, by examining the biomechanical properties of the coated wires [60].

Regarding the antimicrobial activity against *S. mutans* of individual EOs, a better inhibition effect can be appreciated when using OEO and BEO on steel archwires and CEO in the case of molybdenum-based archwires. For all analysed archwires, the ternary mixture (OEO/BEO/CEO) led to inhibition rates and maximum antimicrobial effects.

*S. aureus* is reported to use sortase (Srt) protein in anchoring surface proteins with specific signals in the peptidoglycan layer of their cell membrane 41, thus rendering this protein an essential target of antibacterial. Glucansucrases (glucosyltransferases) are extracellular enzymes produced by the oral floral of *Streptococci*, such as *S. mutans* and *S. sobrinus*. The enzyme plays a crucial role in cariogenesis by allowing the glucan produced to enable the attachment and colonisation of cariogenic bacteria [61]. The last decades have seen advances targeting genes encoding these enzymes in attempts towards developing vaccines and therapeutics that ultimately will stop the disease progression caused by these bacteria [62,63,64,65].

This study established the antibacterial potential of emulsions of essential oils. To further elucidate the molecular dynamics of the interaction between the compounds of these essential oils and key proteins of the bacteria strains (*S. aureus* and *S. mutans*), a computational approach was used to demonstrate this critical revelation. The current study showed binding interactions between the compounds (ligands) and *S. aureus* proteins, sortases A and B, and *S. mutans* glucansucrase proteins. Interestingly, the docking analysis for the *S. aureus* proteins showed the involvement of amino acid residues ASN114, GLN172, LYS175, GLN178, and THR180 for Srt A protein and GLN14, ASN54, ASN63, TYR152, and ARG204 for Srt B in the binding interactions (Figure 10, Figure 11 and Figure 12). These hydrogen bonds were influential in establishing that the ligands exhibited strong molecular interactions with these proteins and hydrophobic and electrostatic interactions. Additionally, for the *S. mutans* protein (glucansucrase), the docking analysis showed the amino acid residues THR305, GLN367, SER368, ALA369, ASP477, HIS587, and GLN592 were critical to the binding interaction. The involvement of ASP477 in glucansucrase could mean that this residue is critical for transglycosylation [66].

Overall, it is fascinating that the compounds with the best binding interaction with the protein mainly belong to the monoterpene oxygenate group. This is not surprising, as Perveen, S. (2019) [67] reported how natural compounds such as terpenes, phenolics, non-flavonoids, flavonoids, and alkaloids and their potential to be exploited during drug discovery processes have recently been gaining attention. 

In addition to the antimicrobial effects of essential oils, an important consideration is how these treatments may influence the frictional resistance of orthodontic archwires. Frictional resistance is a critical factor in orthodontic treatments, particularly during the gap-closing phase, as it directly impacts the efficiency of tooth movement. Although there is limited research directly investigating the impact of EO treatments on the frictional properties of stainless steel and titanium–molybdenum alloy archwires, studies on surface coatings and treatments suggest that changes to surface characteristics, such as roughness and lubrication, can influence friction. For example, surface coatings or treatments that alter the surface roughness of archwires can either increase or decrease friction depending on the material properties and the nature of the coating applied [24,68]. In 2016, a study by Dridi et al. investigated the frictional force between archwires (stainless steel and NiTi) and brackets under dry and lubricated conditions, comparing self-ligating and conventional ligation systems. Five lubricants—human saliva, olive, sunflower, sesame, and Aloe Vera oils—were used to examine their effect on friction. It was found that the friction behaviour in the archwire/bracket assembly was the best under oil lubrication. The enhancement of the frictional behaviour with natural oils was linked to their main component: fatty acids [69]. This suggests that, given their hydrophobic properties, essential oils could similarly affect the frictional coefficient, potentially reducing friction during orthodontic treatment. At the same time, the primary role of essential oils in this study was their antimicrobial effect; their potential to modify frictional resistance warrants further investigation, as the frictional resistance of orthodontic archwires significantly impacts treatment outcomes. Thus, exploring the effect of essential oil treatments on friction in future studies is crucial to understanding their broader implications in clinical practice.

Based on the literature available, essential oils have demonstrated significant long-term antimicrobial and anti-biofilm efficacy. For example, essential oils like eugenol and thyme oil have been shown to reduce biofilm formation and inhibit the growth of bacteria such as *S. aureus*, with specific concentrations leading to nearly 90% reduction in biofilm formation [70,71]. Additionally, the long-term effectiveness of EOs in preventing biofilm formation is supported by their ability to disrupt bacterial cell membranes, thus reducing their ability to form stable biofilms over extended periods [48,70]. These findings suggest that essential oils could be effective in long-term biofilm inhibition. However, further studies are needed to confirm their efficacy on orthodontic archwires under practical, prolonged use conditions.

These findings contribute abundant insights into the mechanism of action of these emulsions via the ligand–protein binding. Ultimately, the results herein will contribute and guide researchers towards rational drug design procedures with more antioxidant and antibacterial activities.

## 4. Materials and Methods

**Figure 13:**
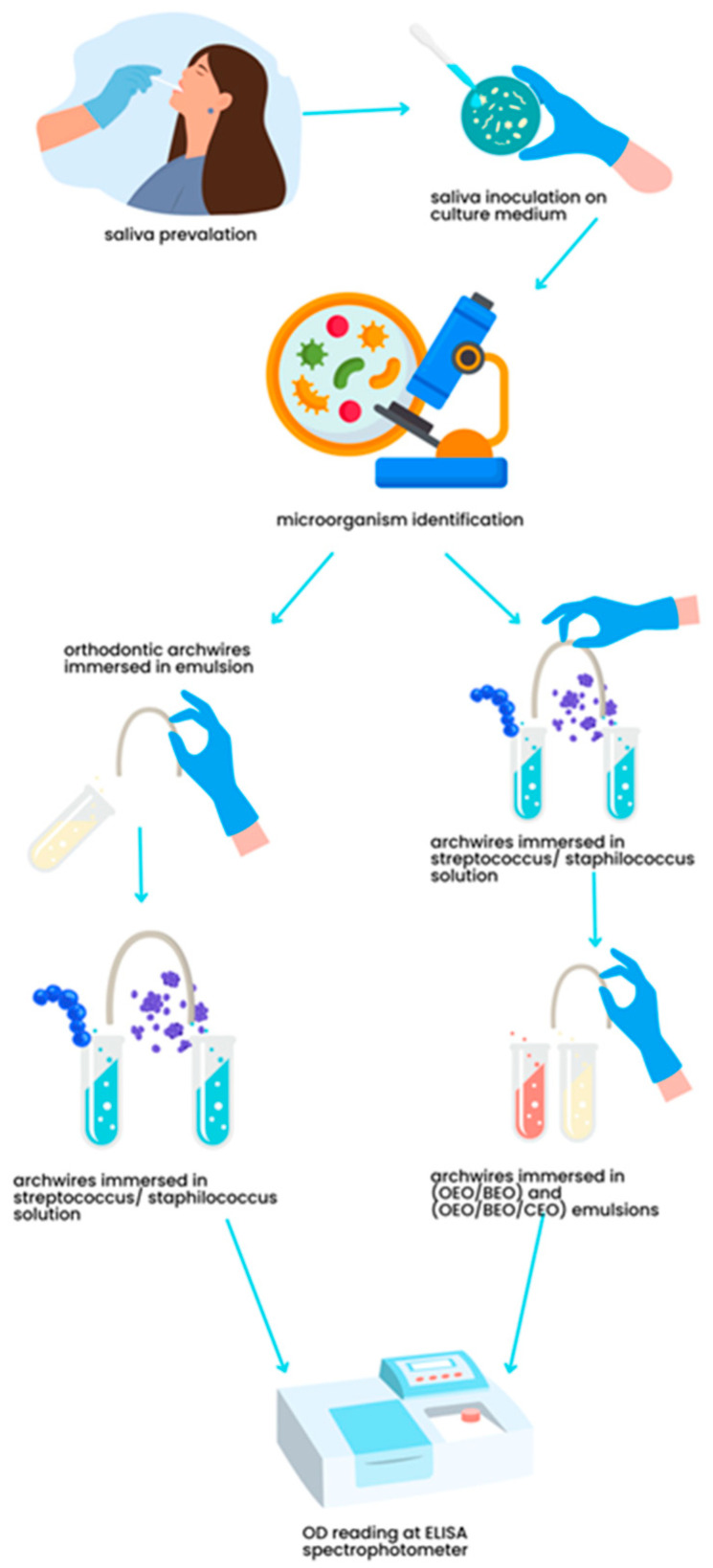
Schematic workflow of the experimental strategy developed for the preventive and curative antimicrobial assays on orthodontic archwires using EOs. Figure created by the authors using Canva.com.

A schematic workflow of the experimental strategy is presented in Figure 13.

### 4.1. Preparation of the Orthodontic Archwires

Five types of orthodontic archwires with a cross-sectional size of 0.019″ × 0.025″ inches were removed from the package the supplier provided (G&H Orthodontics, Franklin, IN, USA) and halved for experimental use. Each wire half’s 1 cm distal ends were cut off and disposed of. Each half of the wire was subsequently cut into pieces of 3 cm.

### 4.2. Preparation of Natural Essential Oil-Based Solutions

**Figure 14:**
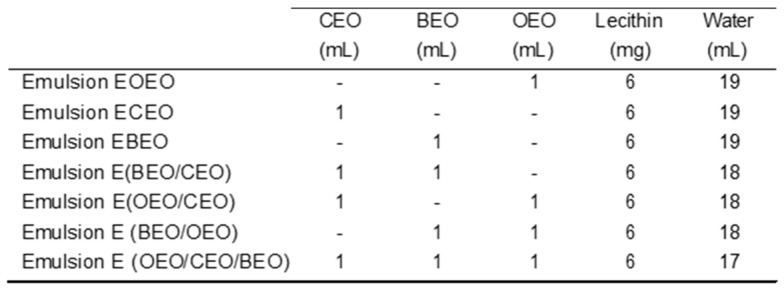
Composition of natural preparations.

The natural preparations based on essential oils were obtained as direct emulsions of oil in water in which the discontinuous phase was the oil globules dispersed in the aqueous phase, as presented in our previous research on the synergistic and antagonistic potential of natural preparations based on essential oils against *S. mutans* from the oral cavity [28]. Natural emulsions with antibacterial properties against *S. mutans* were created using essential oils from cloves (CLEOs), bergamots (BEOs), and orange (OEOs), combined with an emulsifier (lecithin). For each oil, 1 mL in 20 mL of mixture was introduced, thus having a composition of 3.85% each oil in natural preparations. The composition of obtained emulsions is presented in Figure 14.

### 4.3. Isolation of Microbial Strains from Natural Saliva

Natural saliva was taken from a voluntary, healthy donor to isolate existing bacterial strains in the oral cavity according to the methodology described by Salli et al. (2017), with the modification of using human donor saliva in place of artificial saliva [72]. The subject gave his written consent for using saliva and its inclusion in the experiment. The study was approved by the Ethics Committee of the “Victor Babeș” University of Medicine and Pharmacy Timișoara, approval code: Aviz CECS al UMFTVB Nr. 8/30.01.2019.

The saliva was transferred to culture media, and the developed strains were isolated and cultured in Brain Heart Infusion (BHI) broth (Oxoid, CM1135) at 37 °C and, subsequently, passed on BHI Agar for 24 h at 37 °C.

Primary cultures were initiated in BHI broth and BHI broth supplemented with 5% sheep blood to isolate *S. aureus* and *S. mutans* strains. These cultures were then streaked onto BHI agar and BHI agar with 5% sheep blood. For *S. mutans* strains, 5% blood agar was specifically utilised. Bacterial colonies were subsequently classified into species based on cultural characteristics and microscopic smear arrangement.

Gram stains were performed to identify bacterial colonies of interest based on appearance and arrangement in the smear, according to the following methodology: (i) the fixed smear is covered with gentian violet: 1–2 min (wash with tap water); (ii) cover with a solution of Lugol for mordating, 2 min (wash with water); (iii) discoloration with alcohol–acetone for 20 s until no dye is dissolved (wash with water); (iv) recolor with diluted Fuxin/safranin, 2 min (wash off with water); (v) dry the smear and examine it with immersion.

The results are interpreted as follows: (i) Gram + resist fading and maintain their original purple colour; (ii) Gram – undergo discolouration and recolour in red with Fuxin/saffron; (iii) Gram + cocci, arranged in piles (*Staphylococcus* spp.); (iv) Gram+ cocci, arranged in chains (*Streptococcus* spp.).

Optical microscopy was performed using the ×100 objective, and images were processed on an Olympus CX 41 microscope with image capture and data interpretation software.

### 4.4. Preventive Antimicrobial Assay on Orthodontic Archwires Using EOs

The assessment of bacterial growth inhibition was performed using optical density measurements (OD). This technique is widely employed in microbiology and related fields to assess bacterial growth and evaluate the efficacy of antimicrobial agents. The technique involves measuring the turbidity of a bacterial culture at specific wavelengths, typically 600 nm (OD600), using a spectrophotometer. This approach is especially useful for quantifying bacterial growth inhibition, a key parameter in determining the antimicrobial activity of substances. A decrease in OD values compared to controls indicates bacterial inhibition or cell death [73].

The five types of orthodontic archwires were immersed for 1 min (equivalent to one minute of use of a mouthwash solution recommended for prevention) in the solutions of the 7 emulsions presented in Figure 14.

Fresh *S. aureus* and *S. mutans* strains were prepared using BHI broth, being diluted to an optical density (OD) of 0.5 standard McFarland (1.5 × 10^8^ UFC × mL) and evaluated with a McFarland densitometer (Grand-Bio, England). After maintaining contact with emulsions based on EOs, the orthodontic archwires were immersed in isolated cultures of *S. aureus* and *S. mutans*, respectively, and incubated for 24 h at 37 °C. Then, the turbidity of the suspension was read using a 96-well ELISA plate, as described in our previous paper [28]. For the control group, the archwires left in the *S. aureus* and *S. mutans* isolates in BHI were considered without the preventive effect of emulsions.

In order to quantify the bacterial inhibition capacity, the formula for calculating percentage of bacterial inhibition (BIP) was used. The term “Bacterial Inhibition Percentage” (BIP) is commonly used in microbiology to represent the percentage in reducing bacterial growth measured at a specific time point. In the studies of Tyagi et al., 2024, the antibacterial potential of bile salts and inhibition of biofilm formation and cell growth in Pseudomonas aeruginosa and Staphylococcus aureus was appreciated by determining the optical density and the percentage of inhibition as described above [74]. Rekha S. R et al. (2018) determined the antibacterial potential of medicinal plants against Gram-positive (Staphylococcus aureus) and Gram-negative bacteria (Pseudomonas aeruginosa and Klebsiella species) using also the formula of bacterial percentage of inhibition (BIP) [75].

Percentage of bacterial Inhibition (BIP) = (OD of Control − OD of Test)/(OD of Control) × 100%(1)

where ODtest—the optical density (OD) of the sample with emulsion measured at 540 nm, and ODcontrol—the optical density (OD) of bacteria without emulsion.

### 4.5. Healthcare (Curative) Antimicrobial Assay on Orthodontic Archwires Using EOs

To highlight the curative properties of EOs on microbial activity in the oral cavity, the five types of archwires analysed in this study were first immersed in *S. aureus* and *S. mutans* isolates strains grown on culture media according to the methodology described in our previous work [28]. After keeping in contact for 24 h, the orthodontic archwires were immersed in two of the most effective emulsions prepared and selected based on the results obtained in the preventive antimicrobial assay, namely, orange essential oil (OEO)/bergamot essential oil (BEO) and orange/bergamot/clove (OEO/BEO/CEO) essential oil emulsions. Multiple archwires were inserted in the isolate strains for each archwire type to allow removal after a specific timeframe. The experimental design was created to ensure the prelevation of archwires from the isolate stain solution after 48 h to ensure the biofilm formation, after which one archwire was inserted in fresh BHI broth and left for 24 h; the OD was read afterwards to assess the bacterial growth of the strain attached to the archwire, this being considered the control. Another archwire was inserted in the emulsion for one minute and reintroduced in fresh BHI broth for another 24 h. After removing the archwires from the Eppendorf, the broth was introduced into 96-well plates. After each period, the OD was read, and the values were used in Formula (1) o obtain the BGR% and BIR%. The optical density reading of the solutions was carried out per the methodology presented by Alexa et al., 2019 [28]. The orthodontic archwires treated with the ternary emulsion (OEO/BEO/CEO) were subsequently processed for biofilm analysis using confocal microscopy according to the protocol:

Following the incubation period, the archwires were rinsed with distilled water, stained with acridine orange (AO) in acetate buffer (Sigma, St. Louis, MO, USA) for 2 min at room temperature, rinsed in pure water, air-dried at room temperature, and examined using the 100× immersion objective of the Leica DM 2500 fluorescent microscope.

### 4.6. Molecular Docking Assay

#### 4.6.1. Compounds and Proteins Structures Retrieval

The structure of the identified compounds from the GC-MS analysis was retrieved in spatial data file format (SDF) from the Pubchem database (https://pubchem.ncbi.nlm.nih.gov, accessed on 27 November 2024), while the Cryo-EM structure of the proteins in PDB format was retrieved from RCSB protein data bank (http://www.rcsb.org, accessed on 27 November 2024). The LogP value of the compound was computed via the following link: https://colab.research.google.com/drive/185oy5kkLmpQModlhLRfLpc4f9aPcTXdj?usp=sharing, accessed on 27 November 2024.

#### 4.6.2. Molecular Docking

PyRx-Python Prescription 0.8 virtual screening software (https://pyrx.sourceforge.io/, accessed on 27 November 2024) was employed for the docking experiment. In PyRx, the proteins were prepared as autodock molecules and stored in pdbqt format similar to Li et al., 2024 [76]. The compounds were imported to the PyRx and prepared by applying the universal force field (UFF) to minimise the minimum energy for each configuration. Afterwards, the ligands (compounds) were converted to their respective pdbqt format (autodock ligands) in readiness for the docking.

The autodock ligands were docked against the Sortase A and B proteins (PDB ID: 1NG5 and 1T2P) of *S. aureus*, as well as the glucansucrase protein (PDB ID: 3AIE) of *S. mutans*. This was initiated by commanding the Vina wizard to begin the docking process followed by maximising the Auto Grid box for the 1NG5 centre: x, y, z coordinates 17.0927, 14.4958, 2.2474 and dimensions (Angstrom): x, y, z coordinates 41.0190, 49.6809, 47.0061 to cover the entire protein and allow the ligands to find the best binding site in the protein. The same procedure was be adopted for the 1T2P centre: x, y, z coordinates −30.334, 19.716, −0.4542 and dimensions (Angstrom): x, y, z coordinates 46.9210, 39.3621, 41.3839, and 3AIE centre: x, y, z coordinates 193.949, 52.1344, 193.4823 and dimensions (Angstrom): x, y, z coordinates 73.1186, 105.6065, 79.2487.

The binding energies of the interaction that showed the best predicted binding modes to the proteins was computed in PyRx and retrieved in Microsoft excel.

To visualise the docking result and analyse possible receptor–ligand interactions, Discovery studio visualizer (https://discover.3ds.com/discovery-studio-visualizer-download, accessed on 27 November 2024) was used. The 3-D and 2-D interactions were visualised so as to determine the bonding interactions between the amino acid residues of each protein (1NG5, 1T2P, 3AIE) and the ligands (compounds).

Data availability: The authors of this paper will freely provide all data on request.

### 4.7. Statistical Analysis

The mean values and standard deviations for all replicates were calculated using Excel software. One-way ANOVA was performed to analyse differences between means, followed by multiple comparisons using the *t*-test. Differences were considered significant at *p*-values < 0.05.

## 5. Conclusions

Biofilm adhesion is a critical factor in selecting orthodontic archwires for patients at risk of dental caries and periodontal diseases. This study demonstrated that biofilm adhesion and antimicrobial efficacy of essential oil-based treatments depend on the archwire material.

Steel-based orthodontic archwires, particularly aesthetic epoxy-coated stainless steel, exhibited the highest antimicrobial protection against *S. mutans* strains (maximum BIP: 28.82%). For *S. aureus* strains, inhibition effects were observed only in titanium–molybdenum alloy (TMA) orthodontic archwires, with the maximum inhibition rate reaching 29.44% across all tested emulsions.

Conversely, CuNiTi alloys did not exhibit any inhibition effects against *S. aureus* or *S. mutans* strains, regardless of the EO preparation used. Stainless steel alloys showed promising protective effects against *S. mutans*, with positive inhibition rates achieved using mono-, binary, and ternary EO mixtures. However, stainless steel alloys were not effective in preventing contamination with *S. aureus*.

Post-biofilm contamination treatment with EOs revealed that the ternary emulsion (BEO/CEO/OEO) was the most effective against both bacterial strains for four of the five tested archwire materials. CuNiTi alloys, however, showed consistently low inhibition rates for both strains, with BIP values ranging from 4.24% to 7.69% for *S. mutans* and 6.50% to 12.93% for *S. aureus*.

The synergistic effects of the EOs and their antibacterial potential were evident, highlighting their usefulness in reducing oral bacterial growth. Based on the findings, we recommend aesthetic epoxy-coated stainless steel or TMA archwires combined with preventive treatment using the relevant BEO/CEO/OEO ternary mixture for optimal antibacterial protection against biofilm formation.

## Data Availability

The data presented in this study are available on request from the corresponding author.

## Appendix A

Binding energy and interaction between the compounds and *S. aureus* Srt B protein (1NG5).

**S/No**	**Compounds**	**Pubchem ID**	**LogP Value**	**Binding Energy (kcal/mol)**	**Binding Interaction**
1	α-pinene	6654	2.9987000000000013	−5.3	Nil
2	camphene	6616	2.9987000000000013	−4.9	Nil
3	β-pinene	10290825	2.9987000000000013	−5.1	Nil
4	thujene	17868	2.9987000000000013	−5.3	Alkyl: LYS12, LEU13, LYS16, ILE38
5	β-myrcene	31253	3.4750000000000023	−4.4	Alkyl/Pi-Alkyl: LEU67, PHE82, TYR99, ILE153, CYS194, ARG204
6	D-limonene	22311	3.308900000000002	−5.1	Alkyl: LYS12, LEU13, LYS38
7	eucalyptol	2758	2.7441000000000013	−5.1	Alkyl: LEU67, CYS194
8	O-cymene	28567	2.5222200000000004	−5.8	H: TYR152, THR193 Pi-Sigma: TYR99
9	4-carene	530422	2.8546000000000014	−4.9	Nil
10	butanoic acid hexyl ester	17525	2.910000000000001	−4.5	H: TYR152, ARG204 Alkyl/Pi-Alkyl: PHE82, TYR99, CYS194
11	β-linalool	6549	2.6698000000000013	−5.3	H: GLU195 Unfavourable Donor–Donor: ARG204 Pi-Alkyl: TYR99
12	α-caryophyllene	5281520	5.0354000000000045	−6.0	Pi-Sigma: TYR99
13	β-farnesene	15228937	5.425500000000005	−4.7	Pi-Sigma: TYR99 Alkyl/Pi-Alkyl: LEU67, CYS194, ALA194
14	α-terpineol	17100	2.5037000000000007	−5.3	H: GLN14 Alkyl/Pi-Alkyl: ARG7, TYR10, TYR55
15	cinnamaldehyde	637511	1.8987	−5.2	H: LYS78 Pi-Cation: ARG7 Pi-Alkyl: ARG7
16	cinnamyl-alcohol	5315892	1.6921000000000002	−5.2	H: GLU6 Pi-Cation: ARG7 Pi-Alkyl: ARG7
17	cinnamyl-acetate	5282110	2.2629	−5.2	H: ASN63 Pi-Sulfur: CYS194 Pi-Pi T-shaped: TYR99 Pi-Alkyl: LEU67
18	p-eugenol	3314	2.1293	−5.3	Pi-Cation: ARG7 Pi-Pi T-Shaped: TYR55 Alkyl/Pi-Alkyl: ARG7, TYR10
19	eugenol acetate	7136	2.349	−5.7	H: GLN14, ASN54 C-H/Pi-Donor H-Bond: GLU11, GLN14 Pi-Cation: ARG7 Alkyl/Pi-Alkyl: ARG7, TYR10, MET108
20	chavicol	68148	2.1207	−5.2	Pi-Pi T-shaped: PHE71 Alkyl/Pi-Alkyl: LYS12, LEU13, LYS16

## Appendix B

Binding energy and interaction between the compounds and *S. aureus* Srt A protein (1T2P).

**S/No**	**Compounds**	**PubChem ID**	**Logp Value**	**Binding Energy (kcal/mol)**	**Binding Interaction**
1	α-pinene	6654	2.9987000000000013	−4.6	Nil
2	camphene	6616	2.9987000000000013	−5.2	Nil
3	β-pinene	10290825	2.9987000000000013	−4.8	Nil
4	thujene	17868	2.9987000000000013	−5.4	Alkyl: ILE158, VAL166, VAL168, ILE199, VAL201
5	β-myrcene	31253	3.4750000000000023	−5.1	Alkyl: ILE158, VAL166, VAL168, ILE199, VAL201
6	D-limonene	22311	3.308900000000002	−6.1	Alkyl: PRO163, VAL166, VAL168, ILE199, VAL201
7	eucalyptol	2758	2.7441000000000013	−4.8	Nil
8	O-cymene	28567	2.5222200000000004	−6.4	H: ASN114, THR180 Pi-Sigma: ILE199
9	4-carene	530422	2.8546000000000014	−4.8	Alkyl: LYS198
10	butanoic acid hexyl ester	17525	2.910000000000001	−4.7	H: THR180 C-H: 178 Alkyl: ILE158, PRO163, VAL166, VAL168, ILE199, VAL201
11	β-linalool	6549	2.6698000000000013	−4.6	Alkyl: LYS196
12	α-caryophyllene	5281520	5.0354000000000045	−6.1	Nil
13	β-farnesene	15228937	5.425500000000005	−6.0	Alkyl: ILE158, VAL166, VAL168, ILE182, ILE199, VAL201
14	α-terpineol	17100	2.5037000000000007	−6.0	H: GLN178 Alkyl: VAL166, VAL168, ILE199, VAL201
15	cinnamaldehyde	637511	1.8987	−5.2	H: LYS175, GLN178 Pi-Sigma: ILE199 Pi-Alkyl: VAL166, VAL168
16	cinnamyl-alcohol	5315892	1.6921000000000002	−5.7	H: GLN172, LYS175, GLN178 Pi-Sigma: ILE199 Alkyl: VAL166, VAL168
17	cinnamyl-acetate	5282110	2.2629	−6.3	H: LYS175, GLN178 Pi-Donor H-bond: VAL168 Pi-Sigma: ILE199 Alkyl/Pi-Alkyl: VAL166, VAL201
18	p-eugenol	3314	2.1293	−5.9	H: GLN178, THR180 Pi-Sigma: ILE199 Alkyl/Pi-Alkyl: PRO163, VAL166, VAL168
19	eugenol acetate	7136	2.349	−6.0	C-H/Pi-Donor H-Bond: VAL168 Pi-Sigma: ILE199 Alkyl/Pi-Alkyl: VAL166, VAL168, VAL201
20	chavicol	68148	2.1207	−5.6	Pi-Donor H-Bond: VAL168 Pi-Sigma: ILE199 Alkyl/Pi-Alkyl: VAL166, VAL201

## Appendix C

Binding energy and interaction between the compounds and *S. mutans* glucansucrase protein (3AIE).

**S/No**	**Compounds**	**Pubchem ID**	**LogP Value**	**Binding Energy** **(kcal/mol)**	**Binding Interaction**
1	α-pinene	6654	2.9987000000000013	−5.8	Nil
2	camphene	6616	2.9987000000000013	−5.5	Nil
3	β-pinene	10290825	2.9987000000000013	−5.8	van der Waals: LEU433 Pi-Alkyl: TYR916
4	thujene	17868	2.9987000000000013	−5.4	Pi-Alkyl: TYR916
5	β-myrcene	31253	3.4750000000000023	−4.9	Alkyl/Pi-Alkyl: MET614, LYS618, PHE621, PHE866
6	D-limonene	22311	3.308900000000002	−5.7	Alkyl/Pi-Alkyl: LEU382, LEU433, TYR610, PHE907, TYR916
7	eucalyptol	2758	2.7441000000000013	−5.8	Pi-Sigma: TYR916 Alkyl: LEU433
8	O-cymene	28567	2.5222200000000004	−6.3	H: HIS587, GLN592 Unfavourable Donor–Donor: ASN862 Pi-Anion: ASP588
9	4-carene	530422	2.8546000000000014	−5.4	Nil
10	butanoic acid hexyl ester	17525	2.910000000000001	−4.4	H: HIS587 Alkyl/Pi-Alkyl: LEU433, ALA478, PHE907
11	β-linalool	6549	2.6698000000000013	−5.0	Unfavourable Acceptor–Acceptor: ASP477 Alkyl/Pi-Alkyl: LEU382, TYR610
12	α-caryophyllene	5281520	5.0354000000000045	−7.0	van der Waals: LEU433
13	β-farnesene	15228937	5.425500000000005	−5.5	Alkyl/Pi-Alkyl: LEU382, LE433, HIS587, TYR610, TYR916
14	α-terpineol	17100	2.5037000000000007	−6.0	Pi-Sigma: TYR916 Alkyl/Pi-Alkyl: LEU382, LEU433, TYR610, PHE907
15	cinnamaldehyde	637511	1.8987	−5.3	H: THR305, GLN367 Pi-Alkyl: PRO301
16	cinnamyl-alcohol	5315892	1.6921000000000002	−5.3	Unfavourable Donor–Donor: GLN367 Pi-Alkyl: PRO301
17	cinnamyl-acetate	5282110	2.2629	−5.9	H: GLN367, SER368, ALA369 Alkyl/Pi-Alkyl: PRO301, TRP370
18	p-eugenol	3314	2.1293	−5.5	H: HIS587, ASP477 C-H: ASP909 Unfavourable Donor–Donor: ARG475 Pi-Anion: ASP588 Alkyl/Pi-Alkyl: LEU382, TYR610, TYR916
19	eugenol acetate	7136	2.349	−6.1	H: GLN367, SER368, ALA369 C-H: ASP302, THR305 Pi-Anion: ASP302 Pi-Sigma/Pi-Pi T-Shaped: ALA369, TRP370 Alkyl/Pi-Alkyl: PRO301, LEU1062
20	chavicol	68148	2.1207	−5.6	Unfavourable Donor–Donor: ASN862 Pi-Anion: ASP588, ASP909 Alkyl/Pi-Alkyl: LEU433, LEU434, TYR916
